# Supplementary material for: Transcriptomic Analysis Reveals the Potential Mechanism of Cardamine circaeoides Hook.f. & Thomson in Lowering Serum Uric Acid by Reducing Inflammatory State Through CCR7 Target
Source: Int J Mol Sci. 2024 Dec 2;25(23):12967. doi: 10.3390/ijms252312967 (PMC11641652; doi:10.3390/ijms252312967)
Supplement: Supplementary file 1 [file ijms-25-12967-s001.zip › ijms-3283149-supplementary.pdf]

Supplementary Data

Table S1. Mobile Phase Gradient of UPLC-Q-TOF-MS

Table S2. Mass parameters (Sciex Triple TOF 4600 LC-MS)

Table S3. Primers used for quantitative RT-qPCR

Table S1. Mobile Phase Gradient of UPLC-Q-TOF-MS

Time (min)	A%	B%
0～3	0	100
3～10	0～12	100 ~ 88
10～13	12	88
13～30	12～20	88 ~ 80
30～35	20～45	80 ~ 55
35～40	45～95	55 ~ 5
40～42	95	5
42～42.1	95～0	5 ~ 100
42.1～45	0	100

Note: The UPLC instruments were Waters H-Class (Waters Technologies, USA) and AB Sciex Triple TOF® 4600 LC/MS (SCIEX, USA) and Waters CORTECS® UPLC® T3 (2.1 × 100 mm, 1.6 μm). The mobile phase contains acetonitrile (A) and 0.1% aqueous formic acid (B).

Table S2. Mass parameters (Sciex Triple TOF 4600 LC-MS)

MS		MS/MS	Data
TOF mass range	50 ~ 1700	MS/MS mass range	50 ~ 1250
Ion Source Gas 1（psi）	50	Declustering Potential（V）	100
Ion Source Gas 2（psi）	50	Collision Energy（eV）	±40
Curtain Gas（psi）	35	Collision Energy Spread (eV)	20
Ion Spray Voltage Floating (V)	-4500/5000	Ion Release Delay（ms）	30
Ion Source Temperature (°C)	500	Ion Release Width（ms）	15
Declustering Potential（V）	100		
Collision Energy（eV）	10		

Table S3. Primers used for quantitative RT-qPCR.

Genes	Forward (5'–3')	Reverse (5'–3')
GAPDH	CTGGAGAAACCTGCCAAGTATG	GGTGGAAGAATGGGAGTTGCT
IL27	GTTGCTGCTACCTTTGCTTATGG	TGAAGGGTAAGTTCTGTTAGTGAGGG
Inhbe	TACCAGCATAGACAAATCCACTTCA	GAAGATCCTCAGATACAGAGTGGC
CCR7	TGGCAGACATCCTCTTCCTCA	CATAGCGGTCAATGCTGATACAC
CXCR3	GTTTCCTGATGCCCCTTCTAGTC	TGAGGTGACTGACTTGGCTACG
IL12RB1	TTGAGGTGCAGATTTCCCGTT	CAGCCCTGTTTAAGCCAATGTA
CXCR5	CTCAGAGGCAGAAGGCAGTCA	CGAAGGTGTAGAGCATGGGAT
Mstn	AGAGAGGCGAATGTGGAAAAAGA	AAGTCAGACTCGGTAGGCATGGT
GDF5	ACGACTGGATCATCGCACCC	CTACAGCCACAAGATTCCACGAC
